# The configuration of plantar pressure sensing cells for wearable measurement of COP coordinates

**DOI:** 10.1186/s12938-016-0237-3

**Published:** 2016-10-26

**Authors:** Dian Wang, Ping Cai, Zhiyong Mao

**Affiliations:** 1 School of Electronic Information and Electrical Engineering, Shanghai Jiao Tong University, Shanghai, 200240 China; 2 Shanghai Engineering Research Center for Intelligent Diagnosis and Treatment Instrument, Shanghai, 200240 China

**Keywords:** COP estimation, Plantar pressure, Wearable measurement, Pressure sensing cell layout

## Abstract

**Background:**

Wearable measurement of center of pressure (COP) coordinates is the key of obtaining the measurement of natural gait. Plantar pressure insole is the right sensing unit for plantar pressure monitoring for long-term outdoor measurements and the control of walking assisting exoskeleton robot. It’s necessary to study the configuration of pressure sensing cells.

**Methods:**

This study explored the sensing cell configuration for the plantar pressure insole. The data of plantar pressure of walking is collected for layout variants. The RMSE of COP coordinates estimations are used as the evaluation criteria.

**Results:**

The RMSE of COP coordinates decreases from 8.00 to 3.20 mm as the amount of pressure sensing cells increases from 2 to 7. The size of pressure sensing cells contribute to reduce the RMSE of COP coordinates and 7 pressure sensing cells, with the size of 2.0–2.5 cm have the satisfying performance. Adding pressure sensing cell in the heel and hallux area increase the accuracy of estimating COP coordinates.

**Conclusion:**

Comparison results indicate that the configuration of 7 pressure sensing cells has a satisfying measurement performance.

## Background

Center of pressure (COP) is an important indicator for the evaluation of the equilibrium function and gait analysis [1]. COP trajectory can be used to evaluate the adaptation of prosthetic and the rehabilitation progress of the patients with disable lower limbs [2, 3]. COP sway pattern can be utilized to study the influence on equilibrium impacted by visual signals. And it is also closely correlated with the rehabilitation progress of the stroke patients and Parkinson suffers [4, 5].

Six degrees of freedom force platform is the golden standard for estimating COP coordinates [6]. However, the usage of platform is limited within laboratory. As only one or two isolated steps of gait can be measured with the force plate, strictly speaking, force plate is not suitable for the measurement of natural gait [7].

Commercial off-the-shelf pressure insoles, due to their softness and lightness, looks like the alternative for force plate. Paola Catalfamo et al. used the insole pressure sensors to detect the gait heel-strike and toes-off phase [8]. But the typical pressure insole product is actually designed for pressure mapping, it contains nearly thousands of pressure sensing elements and it is not suitable for the control of walking assisting exoskeleton robot. Besides, in the consideration of applying the ambulatory measurement of COP coordinates into the control of walking assisting exoskeleton robot, it is also necessary to reduce the scale the pressure sensing units, as well as the cost. Therefore, the way of arranging discrete pressure sensing cells according to the plantar anatomical areas is usually adopted when estimating ambulatory COP coordinates.

Some studies used three or five pressure sensing cells located under toes, metatarsals and heel to detect gait phase and estimate COP coordinates [9, 10]. A textile-sensor-based in-shoe plantar pressure measure system used six elements to measure indexes such as COP parameters and peak pressure [11].

Each plantar anatomical area of the foot plays its distinct role in the gait phase, different layouts and sizes of pressure sensing cells of the insole will affect the accuracy of COP coordinate estimation. Therefore, the influence of amount, layout and size on COP coordinates estimation is investigated in this study via comparing different configurations to obtain the optimization with relatively small amount and size of pressure sensing cells.

## Methods

**Fig. 1:**
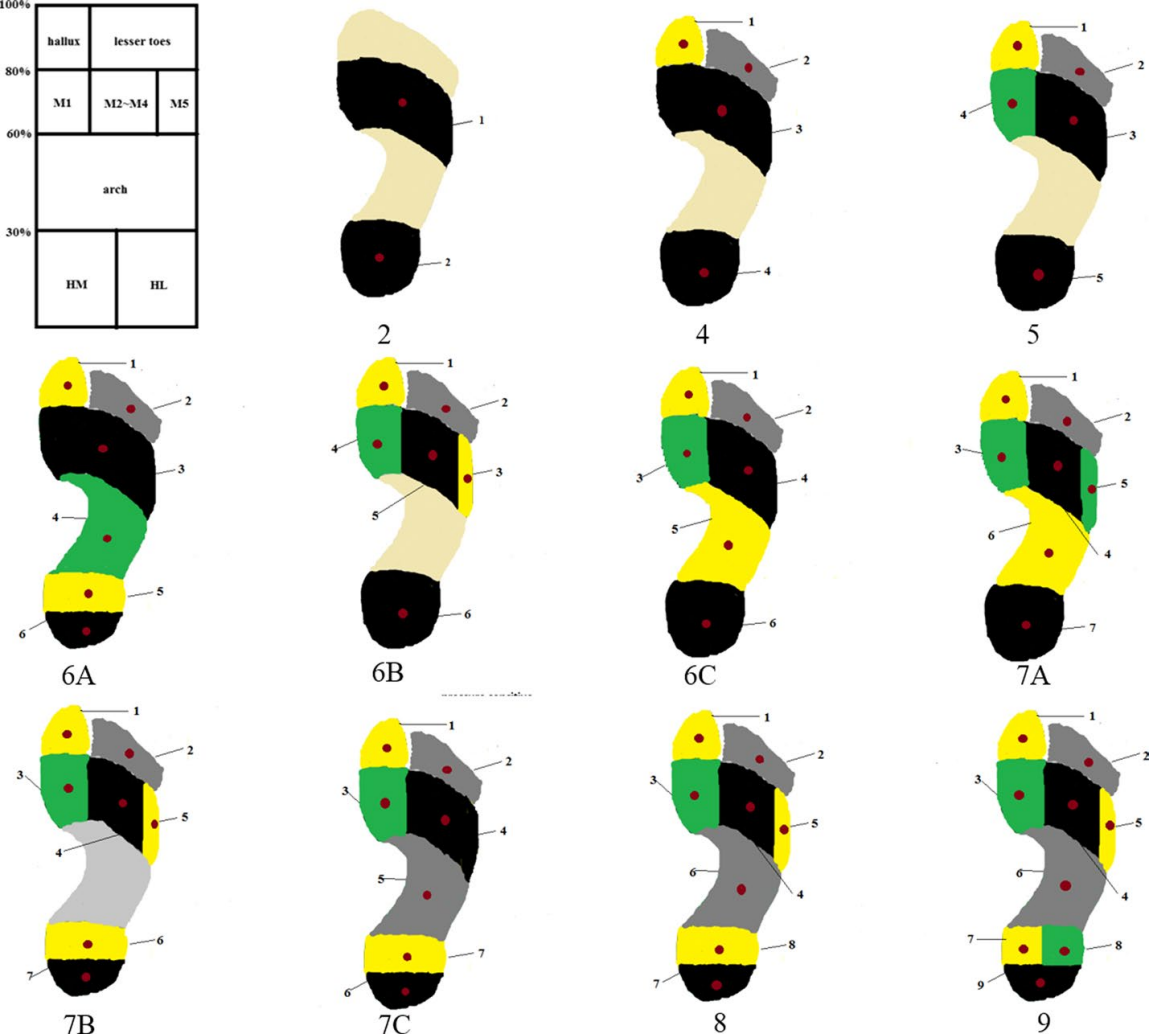
Layout arrangement variant of pressure sensing cells

Figure 1 presents the layout arrangements of the pressure sensing cells. The pressure sensing cells are located in the plantar anatomical partitions. The regional division method adopted is presented in the left top of Fig. 1. The heel can be further divided to anterior heel and posterior heel. The metatarsal region is divided further to three sub-areas as the first metatarsal, the second to the forth metatarsals and the fifth metatarsal by 17:22:12. The toes region is divided into two sub-areas as hallux and the lesser toes by 1:2 as shown in top left of Fig. 1. The spatial resolution of partition dividing is 0.25 mm. Figure 1 shows the layout arrangements of pressure sensing cells and the dark red spots represent sensors which will change size in the following study.

Pressure of each pressure sensing cell is calculated by summing the readout of the cells in the corresponding region of Tekscan 3000E. The pressure range of Tekscan 3000E is 0–2.1 kg/cm^2^ with a spatial resolution of 4 cells/cm^2^. The thickness of Tekscan 3000E is about 0.1 mm and the weight is <10 g. Ten healthy young subjects (6 males and 4 females, age 25.9 ± 3.00 years, weight 61.0 ± 11.6 kg, height 170 ± 8.40 cm) were recruited for this study. All the subjects have no history of motion disorders, none of them suffered from any visible foot deformities. The subjects all gave their written informed consent in accordance with local Ethics Committee of Shanghai Jiao Tong University. Four walking modes: normal walking, walking with the ankle constrained, walking with knee constrained and walking with loads are investigated in this study. The joints are constrained with bandages and protective body gears that are fixed at certain positions to limit the movement range of the joints. Loads are added to subjects by wearing sandbags of 2 kg on the ankles. Subjects need to complete five times 10 step walking trials. Gait data of different configurations of plantar pressure sensing cells is collected in the laboratory and processed with MATLAB 2010b. The layout of Lin Shu’s insole is also investigated in this paper, three pressure sensing cells are placed in the metatarsal region and three pressure sensing cells are placed in the heel region [11]. COP coordinates are estimated through calculating the center of gravity [12] with Tekscan 3000E and each configuration of pressure sensing cells. The estimation errors are calculated by Eqs. (1) and (2).

1 $${\text{RMSE}}_{{{\text{xdirection}}}} = \frac{1}{4} \times \left( \begin{gathered} \left( {\frac{{\sum\nolimits_{1}^{{n1}} {\left( {COP\_X_{{mea}} \left( i \right) - COP\_X_{{ref}} \left( i \right)} \right)^{2} } }}{{n_{1} }}} \right)^{{1/2}} \hfill \\ + \left( {\frac{{\sum\nolimits_{1}^{{n2}} {\left( {COP\_X_{{mea}} \left( i \right) - COP\_X_{{ref}} \left( i \right)} \right)^{2} } }}{{n_{2} }}} \right)^{{1/2}} \hfill \\ + \left( {\frac{{\sum\nolimits_{1}^{{n3}} {\left( {COP\_X_{{mea}} \left( i \right) - COP\_X_{{ref}} \left( i \right)} \right)^{2} } }}{{n_{3} }}} \right)^{{1/2}} \hfill \\ + \left( {\frac{{\sum\nolimits_{1}^{{n4}} {\left( {COP\_X_{{mea}} \left( i \right) - COP\_X_{{ref}} \left( i \right)} \right)^{2} } }}{{n_{4} }}} \right)^{{1/2}} \hfill \\ \end{gathered} \right)$$

2 $${\text{RMSE}}_{{{\text{ydirection}}}} = \frac{1}{4} \times \left( \begin{gathered} \left( {\frac{{\sum\nolimits_{1}^{{n1}} {\left( {COP\_Y_{{mea}} \left( i \right) - COP\_Y_{{ref}} \left( i \right)} \right)^{2} } }}{{n_{1} }}} \right)^{{1/2}} \hfill \\ + \left( {\frac{{\sum\nolimits_{1}^{{n2}} {\left( {COP\_Y_{{mea}} \left( i \right) - COP\_Y_{{ref}} \left( i \right)} \right)^{2} } }}{{n_{2} }}} \right)^{{1/2}} \hfill \\ + \left( {\frac{{\sum\nolimits_{1}^{{n3}} {\left( {COP\_Y_{{mea}} \left( i \right) - COP\_Y_{{ref}} \left( i \right)} \right)^{2} } }}{{n_{3} }}} \right)^{{1/2}} \hfill \\ + \left( {\frac{{\sum\nolimits_{1}^{{n4}} {\left( {COP\_Y_{{mea}} \left( i \right) - COP\_Y_{{ref}} \left( i \right)} \right)^{2} } }}{{n_{4} }}} \right)^{{1/2}} \hfill \\ \end{gathered} \right)$$

where X and Y refer to the A/P and M/L direction, respectively; and refer to COP coordinate obtained with the configured sensing cell pattern; and refer to COP coordinate by Tekscan 3000E; n1, n2, n3 and n4 refer to the total number of samples of aforementioned walking modes, respectively. Gait data of four walking modes aforementioned is grouped together to calculate the total RMSE of COP coordinate estimations of the investigated configuration of plantar sensing cells, utilizing Tekscan 3000E’s result as the truth value.

Six sizes of pressure sensing cells are evaluated in this experiment by combining cells of Tekscan 3000E to obtain the desired size, as the red dots showed in Fig. 1. S1, S2, S3, S4 and S5 represents areas 1.5 cm × 0.5 cm, 1.5 cm × 1.0 cm, 1.5 cm × 1.5 cm, 2.0 cm × 2.0 cm, and 2.0 cm × 2.5 cm, respectively. Pressure sensing cells of fitting the foot contour (S6) are also investigated by combining cells of Tekscan 3000E in the corresponding area, and the division method is shown in the left top of Fig. 1.

## Results

### Result of the influence of the amount and size of pressure sensing cells on the estimation accuracy of COP coordinates

**Fig. 2:**
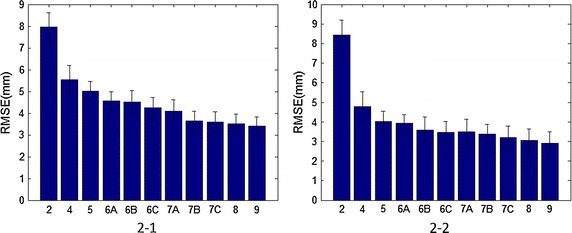
Relation between numbers of pressure sensors and RMSE of COP coordinate estimations. **2-1** refers to A/P direction and **2-2** refers to M/L direction

As it is showed in Fig. 2, when the amount of pressure sensing cells increases from 2 to 4, the accuracy of COP coordinate estimation is improved greatly and when he amount of pressure sensing cells increases from 4 to 5, the accuracy of COP coordinate estimation is improved by 16% in the A/P direction and 9.5% in the M/L direction. RMSE tends to decrease as the amount of pressure sensing cells increases and COP coordinate estimation with six or more pressure sensing cells is improved comparing with those of five or less pressure sensing cells because more pressure sensing cells can cover more plantar anatomical areas involved with walking and running activities. However, as the amount of the pressure sensing cells increases, the improvement of COP coordinate estimation slows down. Tables 1 and 2 also show the statistical difference among different layout arrangement. From layout arrangement of 2 pressure sensing cells to 9 pressure sensing cells, comparisons in A/P direction show that there is no statistical difference among layout 6B, 6C, 7A and 7B, there is also no statistical difference among layout 7C, 8 and 9, however, the statistical differences among other groups are striking. When the amount of pressure sensing cells is more than seven, RMSE of the estimation is not significantly better than seven pressure sensing cells.

**Table 1:** Intergroup statistical differences of COP coordinates estimation RMSE with different numbers of pressure sensing cells (A/P direction)

Group no.	2	4	5	6A	6B	6C	7A	7B	7C	8	9
2	0	1	1	1	1	1	1	1	1	1	1
4	1	0	1	1	1	1	1	1	1	1	1
5	1	1	0	0	1	1	1	1	1	1	1
6A	1	1	0	0	1	1	1	1	1	1	1
6B	1	1	1	1	0	0	0	1	1	1	1
6C	1	1	1	1	0	0	0	0	1	1	1
7A	1	1	1	1	0	0	0	0	1	1	1
7B	1	1	1	1	1	0	0	0	1	1	1
7C	1	1	1	1	1	1	1	1	0	0	0
8	1	1	1	1	1	1	1	1	0	0	0
9	1	1	1	1	1	1	1	1	0	0	0

0 refers to no statistical difference and 1 refers to significant statistical difference (p < 0.05)

**Table 2:** Intergroup statistical differences of COP coordinates estimation RMSE with different numbers of pressure sensing cells (M/L direction)

Group no.	2	4	5	6A	6B	6C	7A	7B	7C	8	9
2	0	1	1	1	1	1	1	1	1	1	1
4	1	0	1	1	1	1	1	1	1	1	1
5	1	1	0	1	1	1	1	1	1	1	1
6A	1	1	1	0	0	1	1	1	1	1	1
6B	1	1	1	0	0	1	1	1	1	1	1
6C	1	1	1	1	1	0	1	1	1	1	1
7A	1	1	1	1	1	1	0	1	1	1	1
7B	1	1	1	1	1	1	1	0	0	1	1
7C	1	1	1	1	1	1	1	0	0	0	0
8	1	1	1	1	1	1	1	1	0	0	0
9	1	1	1	1	1	1	1	1	0	0	0

0 refers to no statistical difference and 1 refers to significant statistical difference (p < 0.05)

Table 3 shows the statistical differences of COP coordinates estimation RMSE among groups with different sizes of pressure sensing cells. Results presented in Table 3 show that larger sensing cells are beneficial for detecting plantar pressure change. Table 4 shows the intergroup statistical differences of COP coordinates estimation RMSE with different sizes of pressure sensing cells. Results show that S5 and S6 have no significant statistical difference.

**Table 3:** RMSE of COP estimation with the different sizes of sensing cells

Size	X (mm) [mean (std)]	Y (mm) [mean (std)]
S1	5.0 (0.5)	3.4 (0.5)
S2	4.6 (0.4)	3.2 (0.5)
S3	4.1 (0.4)	3.1 (0.4)
S4	3.5 (0.3)	2.6 (0.3)
S5	3.1 (0.3)	2.4 (0.3)
S6	2.9 (0.3)	2.3 (0.1)

**Table 4:** Intergroup statistical differences of COP coordinates estimation RMSE with different sizes of pressure sensing cells

	S1	S2	S3	S4	S5	S6
A/P direction						
S1	0	1	1	1	1	1
S2	1	0	1	1	1	1
S3	1	1	0	1	1	1
S4	1	1	1	0	1	1
S5	1	1	1	1	0	0
S6	1	1	1	1	0	0
M/L direction						
S1	0	1	1	1	1	1
S2	1	0	0	1	1	1
S3	1	0	0	1	1	1
S4	1	1	1	0	1	1
S5	1	1	1	1	0	0
S6	1	1	1	1	0	0

0 refers to no statistical difference and 1 refers to significant statistical difference (p < 0.05)

### Result of the influence of pressure sensing elements’ layout on COP estimation

**Fig. 3:**
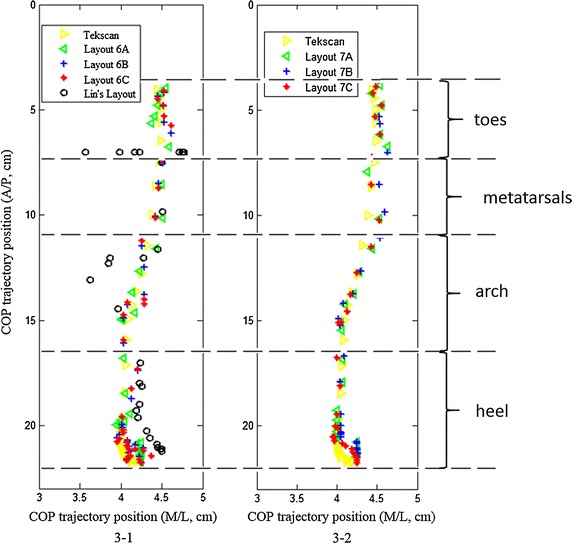
A stride’s COP estimation trajectory with different layouts. **3-1** refers to COP estimation trajectory with six pressre sensing cells and **3-2** refers to COP estimation trajectory with seven pressre sensing cells

The influence of pressure sensing cells layouts on COP coordinate estimation is analyzed through a complete gait. COP trajectories of one step with 6 pressure sensing cells and 7 pressure sensing cells are presented in Fig. 3. Layout adopted by Lin Shu et al. is also included. With the COP trajectory comparisons of a stride showed in Fig. 3, accuracies of COP trajectories with different configurations are studied to for reasonable allocation of pressure sensing cells. Table 5 presents RMSE of every anatomical region with 6 and 7 pressure sensing cells.

**Table 5:** RMSE of COP coordinate in each region

	Heel (A/P, cm)	Arch (A/P, cm)	Metatarsals (A/P, cm)	Toes (A/P, cm)
6A	0.38	0.24	0.40	0.83
6B	0.68	0.40	0.21	0.56
6C	0.66	0.25	0.17	0.29
Lin’s	0.80	0.92	0.24	3.2
7A	0.66	0.25	0.35	0.79
7B	0.60	0.37	0.21	0.76
7C	0.38	0.24	0.17	0.29

	Heel (M/L, cm)	Arch (M/L, cm)	Metatarsals (M/L, cm)	Toes (M/L, cm)
6A	0.39	0.07	0.38	0.34
6B	0.56	0.10	0.32	0.34
6C	0.55	0.07	0.18	0.11
Lin’s	0.72	0.65	0.33	0.80
7A	0.55	0.07	0.19	0.35
7B	0.56	0.10	0.32	0.34
7C	0.39	0.07	0.18	0.11

## Discussion

This study utilizes RMSE of COP coordinates to evaluate the configuration of pressure sensing cells for a COP estimation insole. It has been reported that the mean deviation of COP displacement of health person in A/P or M/L axes is 4 mm [1, 13], we set 4 mm as the criterion to tell the effectiveness of a configuration. RMSE of layout 7C is well <4 mm, as shown in Fig. 2, indicating that seven pressure sensing cells is sufficient for COP estimation in the walking mode. Tables 1 and 2 show that statistical differences among layout 7C, 8 and 9 are not significant, indicating that adding more pressure sensing cells doesn’t contribute much to the estimation accuracy of COP coordinate. For given number of cells and the cell layout, it is not necessary to expend the cell size to the limit, or say to make the cell to fit the plantar anatomic shape, Table 4 shows that the RMSE of S5 is close to that of S6 and they have no statistical difference.

Figure 3-1 shows that when COP coordinates are in the metatarsals region, layout 6C of estimating COP coordinates has the best performance, indicating that it is better to isolate the first metatarsal from the whole metatarsals but not necessary to isolate the fifth metatarsal. It also needs to point out that if six pressure sensing cells are placed as three in metatarsals’ area and three in the heel, which is Lin Shu’s insole layout, they estimate COP trajectory well except the toes-off phase. It is necessary to place at least one pressure sensing cell under the toes to estimate COP coordinates in the gait phase of toes-off.

Figure 3-2 shows that layout 7C, among 6- and other 7-cell-configurations, is the best one whose COP trajectory agrees well with that obtained with Tekscan 3000E. Table 5 shows that the mean RMSE of 7C in heel, arch, metatarsals and toes is lower than that of 7A and 7B, which backs up that subdividing the heel and the metatarsals into two areas is more reasonable for COP coordinate estimation.

To obtain the COP trajectory in a stride, it is necessary to place pressure sensing cells under the four main plantar anatomical areas (toes, metatarsals, arch and heel) and to subdivide metatarsals and toes region into two parts to improve estimation accuracy in M/L axis. Reducing the amount of pressure sensing cells by two orders of magnitude is meaningful for the implementation of wearable measurement of COP coordinates. Reducing the amount of pressure sensing cells is not only beneficial to improve the data throughput and the dynamic performance of the system, but also facilitate the fabrication of weaving customized comfortable pressure-sensing insole.

## Conclusion

This study compares the influence of different layouts, sizes and amounts of pressure sensing cells on COP coordinates estimation. Experiment results indicate that seven pressure sensing cells of 2.0–2.5 cm with the layout 7C will be the optimization of simplifying the wearable system of COP estimation.

## Abbreviations

COP: center of pressure
RMSE: root mean squared error
FSR: force sensitive resistance
A/P: anterior–posterior
M/L: medial–lateral

## References

[1] Hamaoui A, Friant Y, Le Bozec S (2011). Does increased muscular tension along the torso impair postural equilibrium in a standing posture?. Gait Posture.

[2] Schmid M, Beltrami G, Zambarbieri D (2005). Centre of pressure displacements in trans-femoral amputees during gait. Gait Posture.

[3] Mayer Á, Tihany J, Bretz K (2011). Adaptation to altered balance conditions in unilateral amputees due to atherosclerosis: a randomized controlled study. BMC Musculoskelet Disord.

[4] Ruhe A, Fejer T, Walker B (2010). The test-retest reliability of center of pressure measures in bipedal static task conditions: a systematic review of the literature. Gait Posture.

[5] Paillex R, So A (2005). Changes in the standing posture of stroke patients during rehabilitation. Gait Posture.

[6] Cabeza-Ruiz R, Garcia-Masso X, Centeno-Prada RA, Beas-Jimenez JD, Colada JC, Gonzalez LM (2011). Time and frequency analysis of the static balance in young adults. Gait Posture.

[7] Veltink P, Liedtke C, Droog E, van der Kooij H (2005). Ambulatory measurement of ground reaction forces. IEEE Trans Neural Syst Rehabil Eng.

[8] Catalfamo P, Moser D, Ghoussayni S, Ewins D (2008). Detection of gait events using an F-Scan in-shoe pressure measurement system. Gait Posture.

[9] Bamberg S, Benbasat AY, Scarborough DM, Krebs DE, Paradiso JA (2008). Gait analysis using a shoe-integrated wireless sensor system. IEEE Trans Inf Technol Biomed.

[10] Pappas IPI, Keller T, Mangold S, Popovic MR, Dietz V, Morari M (2004). A reliable gyroscope based gait-phase detection sensor embedded in a shoe insole. IEEE Sens J.

[11] Shu L, Hua T, Wang Y, Li Q, Feng D, Tao X (2010). In-shoe plantar pressure measurement and analysis system based on fabric pressure sensing array. IEEE Trans Inf Technol Biomed.

[12] Han T, Paik N, Im M (1999). Quantification of the path of center of pressure (COP) using an F-scan in-shoe transducer. Gait Posture.

[13] Hamaoui A, Le Bozec S, Poupard L, Bouisset S (2007). Does postural chain muscular stiffness reduce postural steadiness in a sitting posture?. Gait Posture.

